# Characterization of the complete chloroplast genome sequence of *Isodon japonicus* (N. Burman) H. Hara (Lamiaceae)

**DOI:** 10.1080/23802359.2022.2123718

**Published:** 2022-09-23

**Authors:** Hao Wang, Yuli Wang, Yang Lu, Shixin Zhu, Jinyong Huang, Caipeng Yue

**Affiliations:** a School of Agricultural Sciences, Zhengzhou University, Henan, China; b School of Life Sciences, Zhengzhou University, Henan, China

**Keywords:** *Isodon japonicus*, Lamiaceae, chloroplast genome, phylogenetic analysis

## Abstract

*Isodon japonicus* (N. Burman) H. Hara (family: Lamiaceae), is a traditional herbal plant from Henan Province, China. In this study, we sequenced the complete chloroplast genome of *I. japonicus* sampled from the Funiu Mountains, Henan Province, China. The total length of the chloroplast genome is 152,298 bp, and has a typical quadripartite structure, including a large single-copy region (LSC) of 83,184 bp, a small single-copy region (SSC) of 17,806 bp and a pair of inverted repeats of 25,654 bp. The chloroplast genome of *I. japonicus* consists of 133 genes, including 88 protein-coding genes, eight rRNA genes, and 37 tRNA genes. There are 34 tandem repeats and 50 simple sequence repeats (SSRs) in the chloroplast genome. The total guanine-cytosine (GC) content of the whole chloroplast genome is 37.6%. Phylogenetic analysis indicated that *I. japonicus* forms a clade with six other *Isodon* species and is closely associated with *I. rubescens*.

*Isodon japonicus* (N. Burman) H. Hara (Hara 1948), known as ‘Shan Su Zi’ in Chinese folk, is a perennial herb that belongs to the genus *Isodon* (family: Lamiaceae). This herb is majorly distributed in Henan, Jiangsu, southern Hubei, southern Gansu, and northern Sichuan in China (Li et al. 1994). *I. japonicus* has long been used in traditional medicine to treat hepatitis, gastritis, tonsillitis, and liver cancer (Di 2015). More than 40 compounds of this species have been isolated and their biological activities have been researched (Jin et al. 2016). Some studies have demonstrated that diterpenoids with antibacterial, anti-inflammatory, and anticancer activities are the key bioactive constituents of *I. japonicus* (Ding et al. 2010; Jin et al. 2019; Xiang et al. 2010). Here, the complete chloroplast genome of *I. japonicus* was reported, and phylogenetic analysis was performed.

Fresh *I. japonicus* samples were collected from Luoyang City, Henan Province, China (N33°42′13.57″, E111°46′36.35″). The voucher specimens were deposited at the Herbarium of Zhengzhou University (ZZU; voucher number: *LY190824MY*, contact person: Shixin Zhu, Email ID: sxzhu@zzu.edu.cn). The modified CTAB method (Doyle and Doyle 1987) was employed to extract total genomic DNA from leaves freshly frozen in liquid nitrogen. Genomic DNA sample was fragmented to a size of 350 bp by sonication. A sequencing library was created using the NEB Next^®^ Ultra™ DNA Library Prep Kit for Illumina (NEB, USA). The complete chloroplast genome of *I. japonicus* was sequenced using Illumina NovaSeq6000 PE150 technology at Novogene Biotech Co. (Beijing,China). After quality control processing of the raw data, 4 GB of data were preserved. The whole complete chloroplast genome was assembled using NOVOPlasty v4.2 (Dierckxsens et al. 2017), and aligned with the sequences of *Isodon* using MAFFT v7.450 (Katoh and Standley 2013). The entire chloroplast genome was annotated using Geneious Prime v2020.2.4 (https://www.geneious.com) and mapped using OGDRAW v1.3.1 (Lohse et al. 2007). Tandem repeats and simple sequence repeats (SSRs) were determined using Tandem Repeats Finder v4.09 (Benson 1999) and MISA v2.1 (Beier et al. 2017), respectively.

The complete chloroplast genome of *I. japonicus* (NCBI accession number MW691144) is 152,298 bp in length, with a typical quadripartite structure, including a large single-copy region (LSC) of 83,184 bp, a small single-copy region (SSC) of 17,806 bp, and a pair of inverted repeat regions (IR) of 25,654 bp. The total guanine-cytosine (GC) content of the chloroplast genome is 37.6%. This genome contains 133 genes (114 single-copy genes), including 88 protein-coding genes (80 single-copy genes), 37 tRNA genes (30 single-copy genes) and eight rRNA genes (four single-copy genes), respectively. Among the annotated genes, 14 genes (eight protein-coding genes and six tRNA genes) contained one intron and two genes (*clpP* and *ycf3*) encompassed two introns. In addition, 34 tandem repeats and 50 simple sequence repeats (SSRs) were detected in the complete chloroplast genome.

31 complete chloroplast genome sequences of Lamiaceae were downloaded from the NCBI GenBank database. *Callicarpa arborea* (MT473738) and *C. macrophylla* (MT473740) were considered as outgroups. Phylogenetic trees were constructed based on the full sequences of the 32 cp genomes employing Maximum Likelihood method (Miller et al. 2010; Stamatakis 2014), and Bayesian inference method, respectively (Ronquist et al. 2012; Zhang et al. 2020). Phylogenetic trees constructed using the two approaches had the same topological structure. The results from the phylogenetic analysis indicated that *I. japonicus* (MW691144) and *I. rubescens* (MW376483) were closely related, and seven *Isodon* species formed a clade. In addition, the topological structure of the phylogenetic tree (including the tribes Ocimeae, Elsholtzieae, Mentheae, and Salvieae) was coherent with the phylogenetic tree of Lamiaceae reported by Zhao et al. (2021). The whole chloroplast genome of *I. japonicus* provides crucial information for the evolutionary studies in Lamiaceae and the development of germplasm resources for this medicinal plant (Figure 1).

**Figure 1.**
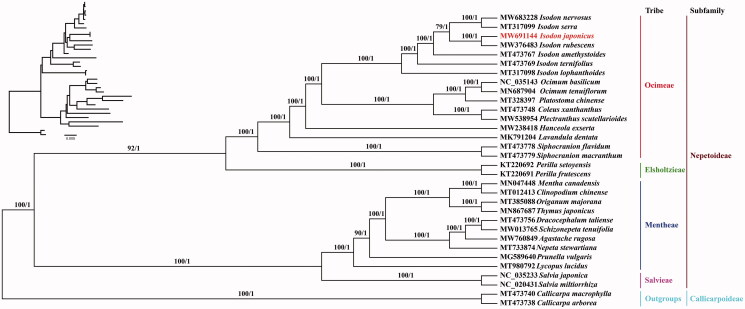
Phylogenetic tree of 32 species based on the complete chloroplast genome by employing the Maximum Likelihood (ML) and Bayesian inference (BI) methods. *Callicarpa macrophylla* and *C. arborea* were outgroups. The numbers above the branches indicate the ML bootstrap values or BI posterior probabilities.

## Ethical approval

The collection of plant material (*Isodon japonicus*) for this study was carried out in strict accordance with the guidelines provided by the herbarium of Zhengzhou University, and the research process for this plant material was carried out in accordance with the guidelines provided by the Chinese regulations. This article does not contain any research on human participants or animals conducted by any of the authors. The specie in this paper is not endangered, protected, or personally owned.

## Author contributions

Caipeng Yue was primarily responsible for the design and financial support of the project, and approved the final version of the paper;

Hao Wang was primarily responsible for the assembly and analysis of sequencing data, as well as the writing and revision of the paper;

Yuli Wang was responsible for sample collection and DNA extraction experiments;

Yang Lu and Shixin Zhu were responsible for sample collection and species identification, and participated in the revision of the paper;

Jinyong Huang was responsible for the financial support and ethical supervision of the project.

## Data Availability

The genome sequence data that support the findings of this study are openly available in GenBank of NCBI at (https://www.ncbi.nlm.nih.gov/nuccore/MW691144), under the accession MW691144. The associated BioProject, SRA, and BioSample numbers are PRJNA786752, SRR17173344, and SAMN23721200, respectively.
